# Supplementary material for: Stacking Model‐Based Classifiers for Dealing With Multiple Sets of Noisy Labels
Source: Biom J. 2025 Mar 12;67(2):e70042. doi: 10.1002/bimj.70042 (PMC11898607; doi:10.1002/bimj.70042)
Supplement: Supplementary file 1 — Supporting Information [file BIMJ-67-e70042-s001.zip › density-based-ensemble-model-main/competitors_real_data/netlab-master/netlab-master/help/netgrad.htm]

Netlab Reference Manual netgrad


# netgrad

## Purpose

Evaluate network error gradient for generic optimizers

## Synopsis

```
g = netgrad(w, net, x, t)
```

## Description

`g = netgrad(w, net, x, t)` takes a weight vector `w` and a network
data structure `net`, together with the matrix `x` of input
vectors and the matrix `t` of target vectors, and returns the
gradient of the error function evaluated at `w`.

## See Also

`mlp`, `neterr`, `netopt`

---

**Pages:**
Index


---

Copyright (c) Ian T Nabney (1996-9)
